# A micro costing analysis of the development of a primary care intervention to improve the uptake of diabetic retinopathy screening

**DOI:** 10.1186/s13012-021-01085-4

**Published:** 2021-02-10

**Authors:** Susan Ahern, Fiona Riordan, Aileen Murphy, John Browne, Patricia M. Kearney, Susan M. Smith, Sheena M. McHugh

**Affiliations:** 1 School of Public Health, College of Medicine & Health, University College Cork, Western Gateway Building, Western Rd., Cork, Ireland; 2 Department of Economics, Cork University Business School, University College Cork, Cork, Ireland; 3 Department of General Practice, Royal College of Surgeons of Ireland, Dublin, Ireland

**Keywords:** Diabetes, Micro-costing, Intervention development, Primary care

## Abstract

**Background:**

The application of economic analysis within implementation science is still developing and the cost of intervention development, which differs markedly from the costs of initial implementation and maintenance, is often overlooked. Our aim was to retrospectively cost the development of a multifaceted intervention in primary care to improve attendance at diabetic retinopathy screening.

**Methods:**

A retrospective micro costing of developing the intervention from the research funder perspective was conducted. It was based on a systematic intervention development process involving analysis of existing audit data and interviews with patients and healthcare professionals (HCPs), conducting consensus meetings with patients and HCPs, and using these data together with a rapid review of the effectiveness of interventions, to inform the final intervention. Both direct (non-personnel, e.g. travel, stationary, room hire) and indirect (personnel) costs were included. Data sources included researcher time logs, payroll data, salary scales, an online financial management system, invoices and purchase orders. Personnel involved in the intervention development were consulted to determine the activities they conducted and the duration of their involvement. Sensitivity and scenario analyses were conducted to estimate uncertainty around parameters and scope.

**Results:**

The total cost of intervention development (July 2014–January 2019) was €40,485 of which 78% were indirect (personnel) costs (€31,451). In total, personnel contributed 1368 h to intervention development. Highest cost activities were the patient interviews, and consensus process, contributing 23% and 34% of the total cost. Varying estimated time spent on intervention development activities by + 10% increased total intervention development cost by 6% to €42,982.

**Conclusions:**

Our results highlight that intervention development requires a significant amount of human capital input, combining research experience, patient and public experience, and expert knowledge in relevant fields. The time committed to intervention development is critical but has a significant opportunity cost. With limited resources for research on developing and implementing interventions, capturing intervention development costs and incorporating them as part of assessment of cost-effective interventions, could inform research priority and resource allocation decisions.

**Supplementary Information:**

The online version contains supplementary material available at 10.1186/s13012-021-01085-4.

Contributions to the literature

- Most studies that consider the cost of implementation start from the point of a fully designed or selected implementation intervention; few studies report the costs of *developing* the intervention.
- Our retrospective micro costing of an intervention to support the implementation of diabetic retinopathy screening addresses this gap, demonstrating how this implementation stage can be costed, and offering insight for other researchers when assigning resources to each of the development steps within their own budgets.
- The findings highlight the significant opportunity cost associated with the time devoted to development, indicating development costs should be considered as part of the assessment of cost-effective interventions.

## Background

Cost is a frequently cited barrier to the implementation and scale up of interventions to improve the uptake of evidence-based healthcare practices (EBP) [1–4]. Despite this, the application of economic analysis within implementation science is limited [5–7]. Where costs are reported, the level of detail on how costs have been identified, measured, and valued is typically sparse [6]. Of the studies that have tried to specify the resources consumed by various implementation activities, most start from the point of a fully designed or selected implementation strategy [8, 9], or focus on resources required for the implementation *process* [10]. Intervention costs can accrue long before implementation begins, yet few studies to date have considered these costs [11–13]. Economic analysis is rarely performed at the early stages such as during intervention development [6]. Few studies included an explicit assessment of the costs of preparatory work such as the costs of developing new processes and training staff, described by the authors as the “hidden costs” of improvement and implementation strategies [5].

A variety of approaches exist for developing and tailoring interventions, and, although there is a lack of consensus on the best approach [14], most are systematic, involving multiple (iterative) stages and input from various stakeholders [15]. The concept of opportunity cost is particularly pertinent; for example, the time given by experts and research teams to the development of an intervention could otherwise be invested in a multitude of projects and endeavours. The cost of intervention development should therefore be regarded as similar to the development cost for new health technologies (e.g. drugs, medical devices, diagnostic tests) and could be considered as part of base-case cost-effectiveness analysis (adjusted for time and expected patient population) [9] so as to inform decisions on whether to adopt the intervention and/or collect further information on the intervention so as to reduce uncertainty surrounding the initial adoption decision based on current information (research priority decision). Ignoring such costs may underestimate and undervalue the cost of intervention development as a distinct and recommended phase. While such costs may be absorbed into “usual activity” in the short run, this can create a free-rider problem [16], which is not conducive to sustainable and efficient intervention development in the long run. This being said, intervention development in an academic context can be considered the creation of new knowledge in the public interest, which, through dissemination (peer-reviewed publications, conference presentations etc.), is made available to the wider research community. In this context, the value of costing the development stage is to raise awareness among different groups (funders, researchers, public) of the level of investment required, to consider this investment in light of the downstream gains, and encourage a more informed assessment of how this work is conducted and funded.

On this point, the level of research funding and the degree to which it reflects the full economic cost (FEC) can differ between countries. For example, in the UK, grants are awarded to academic researchers on the basis of FEC (approx. 80–100%) [17, 18]. In the USA, indirect costs (considered infrastructure or overhead costs not directly related to the project itself) are negotiated and awarded based on cost structures within the institutions [19, 20], while in Ireland funding bodies typically pay a flat rate (approx. 25–30%) towards overhead costs [21]. In the context of this variation and with calls for greater transparency in research funding, it is important to understand and adequately cost the intervention development phase to inform grant applications.

As a case example of a micro-costing approach, our aim was to retrospectively cost the development of the IDEAs (Improving Diabetes Eye-screening Attendance) intervention, a multifaceted implementation intervention based in primary care to improve uptake of Ireland’s national diabetic retinopathy screening programme, Diabetic RetinaScreen. Regular diabetic retinopathy screening (DRS) can prevent and delay diabetes-related blindness through early detection and timely referral for treatment [22]. DRS has also been shown to be cost-effective [23]. However, the success of screening programmes depends on continued high levels of attendance and rates are suboptimal in many countries [24–26]; hence, there has been interest in developing and testing of interventions to improve attendance at diabetic retinopathy screening [27]. While IDEAs was a funded research study, the total value of the award was not based on FEC. The intervention was the subject of a pilot trial conducted in general practices in the Republic of Ireland between July 2019 and July 2020.

## Methods

We conducted the retrospective costing of intervention development from a research funder perspective. While this is a narrower focus than the societal perspective (which would require all costs with a value to society to be considered), it incorporates all direct costs and available indirect costs, for example, personnel time spent on development activities, and cost of office space accounted for in a 25% overhead rate which is the recommended rate for overheads in the national guidelines on conducting economic evaluations and cost analyses. Other indirect costs were excluded as reasonable estimates were unavailable (e.g. researcher training time). We focused on costing each step of the four-step systematic process used to develop the intervention [28].

### Intervention development process

The IDEAs intervention was developed using a stepped approach combining theory, stakeholder involvement and evidence (Table 1) [15, 28]. Stakeholders included people with diabetes, health care professionals (HCPs), and representatives from the national DRS programme. Full details of the intervention development are published [29]. The intervention development work took place in the School of Public Health at University College Cork, Ireland, and drew on data collected at different timepoints, including a primary care audit of DRS uptake in the period July 2014–December 2014 [30]. As per step 1 of the intervention development process, these data were used to identify target behaviours for the intervention, namely patient consent and attendance to the DRS programme, and health care professional registration of patients with the programme. Briefly, the intervention development activities included step 1, ‘identify who needs to do what, differently’, an analysis of audit data; step 2, ‘identify the barriers and enablers to be addressed using theoretical frameworks’, interviews with patients and healthcare professionals were used to identify determinants of uptake; step 3, ‘identify and decide intervention components to address modifiable barriers and enhance enablers’, mapping determinants to behaviour change techniques (BCTs) to develop intervention content, conducting a consensus study with stakeholders to discuss the feasibility, acceptability, and local relevance of BCTs and potential delivery modes, and applying the APEASE criteria (affordability, practicability, effectiveness, acceptability, side-effects, equity) to select the final intervention components and the design of intervention materials and; step 4, ‘how can behaviour be measured and understood?’, developing a logic model of the final IDEAs intervention, representing the inputs, processes, and the causal mechanisms by which the intervention is expected to achieve change.

**Table 1:** Four-step systematic intervention development process and major categories of development activities

Four-step systematic intervention development process			Major category number	Development activity
Step 1	Identify who needs to do what differently		1	Audit and analysis of diabetic retinopathy screening uptake. **Included as part of scenario analysis, scenario A.**
Step 2	Identify the barriers and enablers to be addressed using theoretical frameworks		2	Qualitative patient interviews
			3	Qualitative professional interviews
			4	Coding interviews
Step 3	Identify components to address modifiable barriers and enhance enablers	Step 3a: Identify behaviour change techniques & modes of delivery	5	Mapping process
			6	Identifying effective PPI recruitment strategies for clinical trials. **Included as part of scenario analysis, scenario B.**
		Step 3b: Identify feasibility, local relevance and acceptability of the intervention	7	Consensus process
			8	Populating the APEASE criteria
			9	Intervention design
Step 4	How can behaviour change be measured and understood?		10	Logic model

While the audit (step 1) was not considered an essential part of intervention development, the findings did highlight suboptimal uptake of the DRS programme and distinguished gaps with respect to programme registration, consent for the programme to hold patient contact details and send them an appointment letter, and attendance once they received their appointment [30]. These findings influenced the decision to target both people with diabetes and professionals as part of the intervention [29]. We were able to draw on an existing audit for the current process, but this may not be the case for other intervention developers. In some cases, where data on implementation gaps is not readily available, an audit may form part of the development process. We estimate the additional cost of the conducting the audit, as part of a scenario analysis. The audit of diabetic retinopathy screening uptake in question was conducted in two large primary care centres in the south of Ireland between 2014 and 2015 [30], a number of years before the core intervention development work.

### Implementation costing method

The 4 steps comprised 10 major categories of activities, each of which was costed separately and aggregated to produce a total cost for each of the 4 steps. The costs for each of the 4 steps were then aggregated to produce an overall cost for intervention development. Table 1 lists 10 major categories of activity of which 2 are non-essential activities considered as part of a scenario analysis. Micro costing techniques were applied where all relevant cost components were identified at a detailed level and resource inputs are weighted by unit costs [31]. Data sources included time logs, payroll data, salary scales, an online financial management system, invoices, and purchase orders. Where time log data were not available, research staff who contributed time to the development of the intervention were asked to describe their involvement and recall the approximate time required to complete specific intervention development tasks. Costing was conducted in accordance with guidelines published by the Health Information and Quality Authority (HIQA) in Ireland [32]. Costs are presented in 2019 Euro.

### Personnel (indirect) costs

The involvement of all individuals in each of the major activity categories, the specific nature of the activities they conducted, and the duration of their involvement were documented retrospectively. The name, profession, and grade of the person who conducted these activities, the date of activities, time spent on the activity and a description of the activities were recorded on a spread sheet in Microsoft Excel [33]. The time designated to each of these tasks was mainly self-reported by researchers, with some exceptions, for example, interview audio files, travel time, and documented scheduled meetings. Time was retrospectively gathered from researchers by reviewing calendars, emails, meeting files, personal notes and data collected. Stakeholders’ (people with diabetes, HCPs) involvement and the time they volunteered to the intervention development process were also captured. This is in line with best practice for Patient and Public Involvement (PPI) in research [34]. These resource inputs were identified and reported by researchers who interviewed the stakeholders and conducted focus groups with them. Research staff involved in intervention development included students, research assistants, research support officers, and post-doctoral researchers. Researcher salaries were sourced from the Irish Universities Association Research Salary Guidelines [35]. Academic staff involved in intervention development included the following grades: lecturer, senior lecturer, and professor. All academic salaries, past and present, were sourced from the Human Resources department in University College Cork. The most recent published salary scales are available on the university’s website [36]. Health care professionals involved in intervention development included general practice administrators, general practice nurses, diabetes nurse specialists, general practitioners and ophthalmologists. Health service salaries were sourced from published pay scales of the Health Service Executive in Ireland [37]. In the case of people with diabetes (who participated in both interviews (step 2) and the consensus process (step 3) and patient representatives, occupation data were not gathered. A salary equivalent of average annual earnings, as published by the Central Statistics Office in Ireland [38], was assumed for these individuals. A graphic designer was also employed to design intervention materials and an annual salary was sourced from publicly available data. All salaries used in the base case calculation are available in Additional file 1. In line with established guidelines in Ireland [32], all salaries were adjusted for pay-related social insurance (10.75% of salary), pension costs (4% of salary) and overheads (25% of salary). A 37-h working week was assumed for all individuals and the cost of their time was calculated on a per minute basis. While in Ireland, certain personnel, that is, collaborators with specific expertise who advise on critical elements of the project, are not typically reimbursed as part of the research funding model. This is not the case internationally, for example, in the USA [39]. Therefore, we have incorporated the opportunity cost of their time in the current analysis.

### Non-personnel (direct) costs

Non-personnel direct costs mainly comprised (i) mileage, travel costs, hotel accommodation and subsistence for travelling researchers; (ii) stationery, postage, phone calls and text messages for patient and professional interview recruitment and consensus process recruitment; (iii) catering and room hire for the consensus process and PPI meetings; (iv) transcription services; and (vi) content review of intervention materials by the National Adult Literacy Agency. Where data allowed, itemisation of individual resource items was conducted, and unit costs were applied to value resources. Where data were not available, the actual cost paid (per invoices) was used.

### Sensitivity analysis

As researchers have retrospectively estimated much of the time dedicated to intervention development, in line with national guidance [27], we have conducted one-way sensitivity analysis varying time by ± 10% and ± 20% to estimate the impact on the cost of intervention development. The time spent on all research activities has been subject to this sensitivity analysis with the exception of recorded interviews where the time spent conducting the interviews has been verified by audio and transcript files. In the absence of occupation data for patients with diabetes, PPI participants and patient representatives, a salary equivalent to average annual earnings was used. Given the associated uncertainty, average annual earnings were varied by ± 10% to assess the impact on the total cost of intervention development.

### Scenario analyses

We costed some activities that were not essential parts of the process: (1) activities conducted *prior* to development and not specifically as part of this intervention development process (i.e. conducting and analysing an audit of DRS attendance in primary care), and (2) ‘value added’ activities conducted *as part* of the development of this intervention (i.e. a scoping review of recruitment approaches for PPI). We considered these costs in alternative cumulative scenarios whereby the cost of (A) conducting and analysing the audit, and additionally (B) conducting the scoping review were included. A standalone scenario (C) where one meeting was conducted as part of the consensus process rather than three was also costed. While not essential, these activities contributed to the conception or planning stage of development [15]. The audit confirmed whose behaviour needs to change, while the scoping review was used to inform the approach used to engage relevant stakeholders, specifically, to form our PPI panel. Three consensus groups with PPI-only (meeting 1), PPI and HCP (meeting 2) and HCP-only (meeting 3) were conducted as part of a Study Within a Trial (SWAT) to examine the process and impact of involving different stakeholders in intervention development [39].

## Results

The results presented consist of cost data for all activities forming part of the intervention development process (July 2018–January 2019) including data (interviews with professionals and patients) collected in the period July 2014–January 2015. The total cost of intervention development was €40,485. Table 2 shows the cost data adjusted for inflation, both direct and indirect, for each of the four steps of the intervention development process. Costs range from €22,103 for Step 3 (mapping patient and professional interviews to BCTs, conducting consensus groups, populating the APEASE criteria and designing the intervention) to €229 for step 4 (developing the logic model) to Table 3 shows a detailed breakdown by ‘Major Category of Activity’. Major activity 2 ‘Qualitative patient interviews’ and Major activity 7 ‘Consensus Process’ were the highest cost activities, collectively accounting for 57% of the total cost of intervention development.

**Table 2:** Cost of four-step systematic intervention development process

Step		Indirect cost	Direct cost	Total cost
Step 1	Identify who needs to do what differently	0	0	0
Step 2	Identify the barriers and enablers which need to be addressed using theoretical frameworks	11,100	7,053	18,153
Step 3	Identify components to address modifiable barriers and enhance enablers	20,121	1981	22,103
Step 4	How can behaviour be measured and understood?	229	0	229
**Total cost**		**31,451**	**9,034**	**40,485**

**Table 3:** Cost of major category development activities in systematic intervention development process (base scenario)

Step	Major category number	Development activity	Indirect cost	Direct cost	Total cost
Step 1	1	Audit and analysis of diabetic retinopathy screening uptake	0.00	0.00	0.00
Step 2	2	Qualitative patient interviews	5,350	3,888	9,238
Step 2	3	Qualitative professional interviews	4,251	3,165	7,416
Step 2	4	Coding interviews	1,500	0	1,500
Step 3a	5	Mapping process	2,382	0	2,382
Step 3a	6	Identifying effective PPI recruitment strategies for clinical trials	1611	0	1611
Step 3b	7	Consensus process	12157	1746	13,903
Step 3b	8	Populating the APEASE criteria	2,514	105	2,619
Step 3b	9	Intervention design	1,458	130	1588
Step 4	10	Logic model	229	0	229
**Total cost**			**31,451**	**9,034**	**40,485**

The cost of Major Category 7 ‘Consensus Process’ was €13,903, of which 87% were indirect costs. Researcher time dedicated to the consensus process accounted for 74% (€9081) of these indirect costs. Time spent on the development of PPI meeting materials (€2980, 33%), attending consensus meetings (€1541, 17%), the development of recruitment materials (€1424, 16%) and PPI recruitment (€1,407, 16%) were identified as the largest cost items within researcher time. Both PPI participants’ time and HCP participants’ time spent at the consensus process meetings each accounted for 13% of the indirect costs associated with the consensus process.

### Direct costs

The direct cost of intervention development totalled €9034, comprising 22% of total intervention development cost. Of this cost, 97% was attributable to Major Category 2 ‘Qualitative Patient Interviews’ (43%), Major Category 3 ‘Qualitative Professional Interviews’ (35%) and Major Category 7 ‘Consensus process’ (19%). These costs largely included travel costs for interviews and professional transcription of interviews. The largest direct cost items for the consensus groups activity was room hire (€503) and catering (€330) for the consensus meetings and transcription services (€321). A full detailed breakdown of each major category of development activity is contained in Additional file 2.

### Personnel/indirect costs

Indirect costs (personnel) totalled €31,451, representing 78% of total cost of intervention development. In total, personnel contributed 1368 h to intervention development. Adjusted salaries for those who contributed time for intervention development ranged from €0.19 per minute for a student on placement to €1.47 per minute for an ophthalmologist. Research assistants contributed most time to intervention development, 877 h, representing 57% of total time spent on intervention development. Students on paid placement were the second highest contributors (160 h), representing 12% of the total time, followed by people with diabetes who participated in interviews (step 2) and the consensus process (step 3) (120 h, 9% of total time). In cost terms, research assistants comprised 57% (€18, 051) of total indirect cost, followed by people with diabetes who participated in interviews and the consensus process at 8.9% (€2800), professors at 7.0% (€2213) and students at 5.8% (€1829). The cost of time contributed by expert personnel (i.e. professors, lecturers) not directly funded by the grant, was €4451 (14.2% of the total indirect costs).

### Sensitivity analysis

Varying estimated time spent on intervention development activities by + 10% increased total intervention development cost by 6% to €42,982. The total hours spent on intervention development increased from 1368 to 1485 and total indirect costs increased from €31,451 to €33,948. Conversely, varying retrospectively estimated time spent on intervention development activities by − 10% decreased total intervention development cost to €37,989. Total hours spent on intervention development decreased from 1368 to 1251, and total indirect costs decreased from €31,451 to €28,955. A ± 20% variation in time spent on intervention development resulted in total intervention development costs of €45,478 and €35,492, respectively. Varying average annual earnings by + 20% increased total indirect cost by just 2% or €649.

### Scenario analyses

The total cost of conducting and analysing the audit was €22,893 with the cost of the audit comprising 97% and analysis 3%, bringing the total cost of intervention development to €63,378 (scenario A). Indirect costs for the audit totalled €18,778 (82%), 92% of which was attributable to the researcher’s time spent on conducting the audit. With the scoping review also included (€1169), the total cost of intervention development rose to €64,547 (scenario B). In a standalone scenario (C) where one (mixed) meeting was conducted as part of the consensus process rather than three meetings, the total cost of this activity was reduced by 27%, costing €10,209 rather than €13,903, bringing the total cost of intervention development to €36,792, a reduction of 9%.

## Discussion

This detailed costing of the development of an implementation intervention to increase attendance at diabetic retinopathy screening has yielded a total cost estimate of €40,485, 78% of which is attributable to the time contributed by researchers, patients, PPI participants and healthcare practitioners to the various development activities. These results highlight that intervention development requires a significant amount of human capital input, combining research experience, patient experience and expert knowledge in relevant fields.

### Comparison with existing studies

As evident from the few studies which have costed both the direct and indirect costs of intervention development, the cost varies depending on the nature of the intervention and the approach to development. Our findings align with existing studies, which also reported most costs were attributable to personnel [40], albeit the total cost varied substantially in line with approach to development (i.e. size of the team and type of personnel involved) and the nature of the intervention (i.e. extent of media production and external expertise required). For example, using a similar development process to IDEAs, Mortimer et al. [13] developed an intervention (GP workshops in person or via DVD recording, supplemented with information packs) to support the implementation of clinical guidelines for lower back pain in general practice, reporting total costs of $83,456. Their approach involved substantial work with stakeholders, including the formation of GP advisory groups, focus groups, and subsequent analysis and interpretation. Most costs (66%) were attributable to person-hours contributed by the administrative/research personnel responsible for coordinating and conducting the development work. In comparison, Lairson and colleagues [40] estimated the cost of developing a tailored interactive computer programme to support uptake of colorectal cancer, to be $328, 866 [8], with personnel costs contributing about 69% of total costs (researchers (46%), video and software developers (24%)). Schuster et al. [41] who developed a tailored health literacy intervention (including educational DVD, and guidebook) delivered by community health workers to improve uptake of cervical and breast cancer screening, estimated the cost of this process to be $121,817, with 84% attributable to staff salaries, including researchers, programmers, actors, graphic designers and a photographer/videographer. Similar to our approach, these existing studies costed development using financial records, receipts and invoices [13, 40, 41], administrative records (e.g. details of the venues used and number of participants) [13, 41] and staff wages [13, 40, 41]. Mortimer et al. [13] retrospectively consulted research project staff to obtain information on the process, using this to estimate the proportion of whole time posts spent on development. In contrast, Lairson et al. used weekly staff time logs as the work progressed, incorporating reminders to prompt accurate logs, or, when not maintained, to estimate time retrospectively (approx. 20%) [40].

### Implications

Given the substantial time committed to intervention development, this step should not be overlooked and instead should be considered and costed as part of the economic analysis of new interventions. Ideally, this would inform appropriate funding for such activities and provide estimates for opportunity costs and ensure sustainable development of high-quality interventions. Emphasis on conducting economic evaluations earlier in the intervention lifecycle is increasing [4, 42]. In particular, rather than only considering costs at the full trial stage, it is considered good practice to collect relevant data and understand costs at the development and feasibility stages [42] facilitating informed decisions at this point. We found costing from the funder perspective to be most practical and appropriate at the development stage. However, a broader perspective would be more relevant for in full economic evaluation to determine cost-effectiveness as part of a definitive trial. That is, taking account of all costs and potential outcomes with a value to society (e.g. avoided productivity losses, blindness, premature death). In theory, a societal perspective should be adopted, though we acknowledge in practice national guidelines need to be followed, which often only require a health care provider perspective.

In terms of approaches to incorporate development costs, studies which have costed the development stage, typically conducted analyses both with and without these costs included, in the former scenario amortising (i.e. gradually writing off or spreading) these costs [13, 40] over the number of individuals expected to utilise the intervention [13, 40], and over the expected life of the intervention [40], or evaluating costs and outcomes over different time horizons which allowed for development time and costs [41]. Incorporating development costs has the potential to impact on the findings on cost effectiveness, as illustrated by Mortimer and colleagues, who found their intervention to support guideline uptake was more effective *and* more costly than standard approach to guideline dissemination, but only when development costs were included. Unlike research and development within the pharmaceutical industry [43], the cost of developing interventions to support implementation cannot be recouped through profits, protected by patents. However, as outlined by Mortimer et al., these costs might be set aside as ‘sunk costs’ when there is potential for repeated use in other settings and among other populations [13]. The aim of health services interventions, typically developed through public funding (i.e. research grants and supported by public sector academics), is not to profit but to improve patient outcomes. However, interventions should be still cost-effective to ensure such funding is allocated efficiently and effectively, and to aid decisions about how best to utilise it. The costs of development should still be considered by those developing and trialling such interventions so as to inform research priority decisions, particularly early in the intervention’s lifecycle, even if these costs are not included in the final CEA for the health care funder who considers whether to adopt the intervention or not. For example, information about the substantial investment required to develop interventions should be factored into decisions about whether to develop de novo interventions or use existing interventions, potentially saving resources. However, interventions introduced into different contexts may have different effects [44]. Even if an existing intervention can be utilised, time and effort will still need to be invested to adapt it for a different context. In their systematic review to inform guidance on adaptation, Movsisyan et al. identified 11 key adaptation steps, which included conducting a population needs assessment and obtaining stakeholder input [44]. Although we did not adapt an existing intervention, our findings illustrate that even within an area with numerous existing interventions (i.e. enhancing DRS attendance [45]), and with existing evidence to draw on, following a recommended systematic process to enhance the ‘fit’ with the health care context still requires substantial resources.

The variation in development costs across existing studies reflects the fact there are several different ways to develop interventions with no single best approach. O’Cathain and colleagues, through systematically reviewing the development literature, identified 8 different types of approaches with 18 actions to be considered within those, including deciding who to involve in the development process, and whether to prioritise working with stakeholders or focusing on theory [15]. One of the high cost activities was the patient and professional interviews; time spent on qualitative professional interviews with HCPs contributed 18% (€4251) to the total cost of intervention development. Though conducted prior to the development work, we included these interviews in our core development costs as a key part of development is understanding the barriers and facilitators to the behaviour of interest. Often this takes the form of interviews [46, 47]. It is important to note these interviews were conducted as part of a broader evaluation to understand the implementation of the national clinical programme for diabetes in Ireland, including the establishment of a national DRS programme [48]. Arguably, the time and cost required to conduct these interviews may have been shorter if the topic was restricted solely to the DRS programme. Though a previous systematic review of barriers and facilitators to DRS attendance was available [49], the lack of interviews in the Irish setting meant it was possible that this review might risk missing context-specific barriers in our setting. Furthermore, the results of this previous work enabled the research team to consider known barriers and facilitators and make informed decisions during intervention development*.* This raises an important point, namely that costing development, and raising awareness of such costs, should not prompt developers to only engage in processes which can be streamlined, or which are deemed most efficient. It is important to consider which development approach is most appropriate and feasible given the healthcare context, stakeholder expectations and resource constraints. We used what O’Cathain and colleagues have classified as a stepped theory and evidence based approach to development. Others such as partnership approaches which comprise greater involvement from end-users, often with an emphasis on co-creating knowledge, can be less linear in nature [15] and may be less time efficient. However, there could be downstream gains from using such approaches, including the development of an intervention which is a better ‘fit’ for the context and involves less adaptations and changes as the process moves from feasibility to efficacy studies. If more and different development approaches were costed and considered in light of the broader project (presented alongside a CEA for example), this would yield further insights about the best development approach for the given context.

Our scenario analyses highlight alternative approaches in the development of future interventions. We considered non-essential and ‘value-added’ costs in different scenarios, highlighting these activities in the context of their contribution to the total cost of the development of this intervention, demonstrating how costs can vary depending on status of prior work and approach adopted. Firstly, had the existing audit utilised for step 1 been necessary for the development process, for example, at the conception and planning stage [15] to identify the problem and whose behaviour needs to change, it would have contributed a considerable proportion (36%) to the overall cost of intervention development. The process of manually sorting through patient health records to identify non-attenders at diabetic retinopathy screening is a labour-intensive activity. In other instances, a rapid or systematic review has been conducted to identify who needs to do what differently [22]. A smaller scale audit or an expert consultation may also be an alternative to a full audit. Secondly, the scoping review conducted to identify effective recruitment strategies for PPI in clinical trials (part of step 3a), while considered a non-essential activity in the development of the intervention, was a valuable contribution to the conception and planning stage (18) given one key action is to establish groups to guide the development process and consider how to engage relevant stakeholders. The review contributed to the development of an informed recruitment strategy for PPI. At a cost of €1169, this activity would have contributed just 3% to the total cost of intervention development. Thirdly, had the consensus process been limited to just one mixed meeting, the total costs of development (excluding the audit and scoping review) would have been reduced by 9%. However, in a separate study we found that group composition influenced group dynamics and consequently contributions on the intervention ‘fit’ with the local context [50].

### Strengths and limitations

Our study adds to the very limited body of literature in a research area that has received little attention to date but that should be regarded as a fundamental aspect of the development of any intervention. However, the cost estimate has a number of limitations. First, the results may not be generalisable to other jurisdictions given that Irish wage rates, overhead rates, pension contribution and social insurance rates may not be applicable elsewhere. However, the exercise clearly demonstrates that a significant portion of intervention development costs can be accounted for with personnel time, 77% in our study. The development of an intervention requires a large amount of human capital, with expert knowledge in fields such as behavioural science and public health, that could otherwise be invested in a multitude of projects and endeavours. In that regard, our study can be compared to a number of other studies that have been conducted to cost the development of interventions to increase attendance at cancer screening, where the cost attributable to personnel has varied from 67% [44] to 74% [51]. Second, much of the data on research time spent developing the IDEAs intervention was retrospectively gathered from researchers during the intervention development process. Given that the costing exercise included activities that commenced with the audit of DRS uptake in June 2015 and concluded with development of the logic model almost 4 years later (May 2019), recollection of time spent contributing to the various steps may be subject to recall bias, an issue other studies have also cited [40, 41]. We tried to account for this by conducting sensitivity analyses accounting for varying the time spent on different activities. While we accept that accurately estimating time is difficult, we are confident we have identified and used accurate records to compile other non-personnel costs. Co-authors involved in all stages of development (2015–2019) (FR and SMH) were able to identify and draw on email records, calendars and meeting minutes to inform estimates, and provide oversight over the costing process. For the later development steps, the lead author SA consulted with more than one researcher for certain steps (e.g. preparation of a questionnaire for the consensus process involved FR and two RAs) to verify the time and resources involved, including cross-checking emails and notes. Most of those involved in the core development work (FR, SMH, JB, SS, PK, AM) are co-authors and had the opportunity to review costs. Regular meetings were held between the lead author SA, and AM, FR and SMH to review the cost breakdown and discuss time estimates and any gaps. Having undertook this process, we would advise different approaches to documentation which would make costing easier and more accurate than retrospective accounts, for example using weekly staff logs as the work progressed in line with the approach used by Lairson et al. [40]. Third, our decisions about what was essential and non-essential parts of the development process, was dictated by our approach to development, a theory and evidence-based process which has been widely used. However, elements of our decisions were considered subjective. In general, if we felt the intervention could have been developed without using a specific method or step, then we considered that part non-essential. So, while we felt the target behaviours could be identified without an audit (step 1), we knew step 2 would always involve a process to develop an understanding of barriers and facilitators, typically using interviews, and so this part was considered essential. While step 3 should identify the feasibility, acceptability and local relevance of the intervention, we chose to conduct consensus process but could have modified elements of this (i.e. fewer meetings, presenting an intervention plan to critique rather than options for intervention content). As we were aware of the subjective nature of our decisions and given there is no single best approach to develop interventions, we included the scenario analyses to make the costs as transparent as possible. Lastly, for reasons of confidentiality and data protection, the occupations of PPI participants were not sought and collected during intervention development. An assumption was made that average annual earnings for the relevant year, as published by the Central Statistics Office in Ireland [38], should be used as a salary proxy for patients who participated in interviews, PPI participants and 2 contributors whose stated profession did not enable us to establish a salary. While accepting this as a limitation it should be noted that the number of hours contributed to intervention development from these participants represented 9% of the total time spent on intervention development. We also conducted sensitivity analysis to assess the impact of potentially higher salaries. Average annual earnings were varied by + 20% and this increased the total intervention development cost by just 2%.

## Conclusions

We have identified, measured and valued the costs relevant to each of the four steps of the development process, providing clarity on how the cost burden is distributed throughout the process. Our work highlights the extent of human resources required in the development of implementation interventions, the opportunity costs associated with the time devoted to intervention development and the aspects of intervention development work that demand greatest human resources. We believe this work should provide insight to fellow researchers involved in intervention development to consider how resources might be most efficiently deployed for intervention development. The results should also provide context and insight for other researchers who follow the stepped systematic intervention development process in considering how best to assign resources to each of the steps within the limits of their own budgets. Following the conclusion of the pilot trial of the IDEAs intervention, the results of this micro-costing will be used, alongside the results of an economic analysis of the cost of delivering the intervention in general practice, to inform future decisions about moving to a definitive trial of the IDEAs intervention. In proceeding to a definitive randomised controlled trial, the results from this micro costing could also be used to determine if the level of investment in developing the intervention is deemed cost-effective. With ever increasing demands on limited health resources, on-going budget constraints and the need to ensure that resources are allocated efficiently, the process of costing intervention development should be an integral part of the economic analysis of all new health technologies.

## Supplementary Information

**Additional file 1.** Master cost items.

**Additional file 2.** Salaries.

## Data Availability

Not applicable.

## Abbreviations

HCPs: Health care professionals
DRS: Diabetic retinopathy screening
EBP: Evidence-based healthcare practices
FEC: Full economic costing
IDEAs: Improving Diabetes Eye-screening Attendance
HIQA: Health Information and Quality Authority
PPI: Patient and Public Involvement
SWAT: Study Within a Trial
